# Comparable Outcomes Across 4 Hamstring Tendon Graft Configurations in ACL Reconstruction, Despite Increased Knee Laxity With Gracilis Tendon Harvesting

**DOI:** 10.1177/23259671251363585

**Published:** 2025-08-26

**Authors:** Riccardo Cristiani, Christoffer von Essen, Eric Hamrin Senorski, Riccardo D′Ambrosi, Camilo P. Helito, Kristian Samuelsson, Anders Stålman

**Affiliations:** † Department of Molecular Medicine and Surgery, Section of Sports Medicine, Karolinska Institutet, Stockholm, Sweden; ‡ Stockholm Sports Trauma Research Center (SSTRC), FIFA Medical Centre of Excellence, Sophiahemmet, Valhallavägen 91, 114 86, Stockholm, Sweden; § Department of Health and Rehabilitation, Institute of Neuroscience and Physiology, Sahlgrenska Academy, University of Gothenburg, Gothenburg, Sweden; ‖ IRCCS Ospedale Galeazzi, Sant′Ambrogio, Milan, Italy; ¶ Universita′ degli Studi di Milano, Dipartimento di Scienze Biomediche per la Salute, Milan, Italy; # Hospital Sírio Libanês, São Paulo, SP, Brazil; ** Grupo de Joelho, Instituto de Ortopedia e Traumatologia, Hospital das Clínicas HCFMUSP, Faculdade de Medicina da Universidade de São Paulo, São Paulo, SP, Brazil; †† Department of Orthopaedics, Institute of Clinical Sciences, Sahlgrenska Academy, University of Gothenburg, Gothenburg, Sweden; Investigation performed at Stockholm Sports Trauma Research Center, Karolinska Institutet, Stockholm, Sweden

**Keywords:** anterior cruciate ligament, ACL reconstruction, hamstring tendons, knee laxity, revision ACLR, muscle strength, subjective knee function

## Abstract

**Background:**

There is a lack of studies that have thoroughly compared subjective and objective outcomes in patients undergoing anterior cruciate ligament reconstruction (ACLR) using different hamstring tendon (HT) graft configurations.

**Purpose/Hypothesis:**

The purpose of this study was to compare anterior knee laxity, isokinetic knee extension and flexion strength, single-leg hop (SLH) test performance, subjective knee function, and the 5-year revision surgery rates between patients who underwent ACLR using 4 HT graft configurations. It was hypothesized that there would be no significant differences in the outcomes examined between the groups.

**Study Design:**

Cohort study; Level of evidence, 3.

**Methods:**

Patients ≥16 years without concomitant ligamentous injuries who underwent primary ACLR with an HT autograft at Capio Artro Clinic, Stockholm, Sweden, between January 1, 2005, and December 31, 2018, were identified. Anterior knee laxity was evaluated using the KT-1000 arthrometer (134 N) both preoperatively and at 6 months postoperatively. Isokinetic knee extension and flexion strength, along with SLH test performance, were evaluated 6 months postoperatively. Subjective knee function was evaluated using the Knee injury and Osteoarthritis Outcome Score, which was collected preoperatively and at 1, 2, and 5 years postoperatively. Revision ACLRs performed at any institution in Sweden within 5 years of the primary surgery were captured using the Swedish National Knee Ligament Registry.

**Results:**

A total of 5673 patients (55.7% male) were included: tripled semitendinosus tendon (ST3), 245 patients; quadrupled semitendinosus tendon (ST4), 4359 patients; doubled semitendinosus + doubled gracilis tendon (ST-G4), 915 patients; and quadrupled semitendinosus + doubled gracilis tendon (ST-G6), 154 patients. Preoperatively, the groups showed no significant differences in anterior knee laxity. Postoperatively, the ST-G4 and ST-G6 exhibited greater mean anterior side-to-side (STS) laxity and increased STS laxity based on the International Knee Documentation Committee examination form, with fewer patients showing STS laxity ≤2 mm and more patients having STS laxity between 3 and 5 mm and >5 mm (*P* <.001). No significant differences were found between the groups in terms of extension and flexion strength or SLH test performance. Regarding subjective knee function, the only significant differences between the groups, though not clinically relevant (<8-10 points), were observed in the preoperative Pain and Quality of Life subscales of the KOOS, as well as in the 1-year Symptoms subscale. The 5-year revision ACLR rates were as follows: ST4, 4.7% (207/4359); ST3, 5.3% (13/245); ST-G4, 3.7% (34/915); and ST-G6, 5.8% (9/154). The hazard of revision ACLR within 5 years of primary surgery in the ST3, ST-G4, and ST-G6 groups was not significantly different from that of the ST4 group (reference group).

**Conclusion:**

All 4 HT graft configurations (ST3, ST4, ST-G4, and ST-G6) yielded comparable outcomes in isokinetic knee flexion and extension strength, SLH test performance, subjective knee function, and the hazard of revision surgery after ACLR. The groups that underwent gracilis tendon harvesting (ST-G4 and ST-G6) exhibited increased anterior knee laxity at 6 months compared with the groups that did not (ST3 and ST4). However, the clinical significance of this finding remains uncertain, as the greater knee laxity did not correlate with subjective knee function or revision ACLR.

Hamstring tendons (HTs) are a commonly used autograft worldwide for anterior cruciate ligament (ACL) reconstruction (ACLR),^2^ with multiple possible graft configurations. The semitendinosus tendon (ST) can be used alone as a tripled (ST3) or quadrupled (ST4) graft or combined with the gracilis tendon as a doubled ST + doubled gracilis (ST-G4) or quadrupled ST + doubled gracilis (ST-G6) to increase graft length or diameter. However, there is a lack of studies that have comprehensively compared all 4 HT graft configurations in terms of both subjective and objective outcomes after ACLR, making it difficult to favor one configuration over another.

Another unresolved issue is the effect of gracilis harvesting on knee flexion strength, as studies have reported conflicting findings—some indicating greater deficits after gracilis harvesting,^18,21,24,34^ while others found no significant effect.^5,7,20,29^ Symmetrical knee strength is considered important for a successful return to sport after ACLR^11,41^ and is associated with a lower risk of reinjury.^16,23^

Finally, the effect of gracilis harvesting on revision ACLR remains insufficiently studied. Hamstring contraction helps protect the ACL by controlling anterior tibial translation and internal tibial rotation,^4,26,28^ potentially reducing the risk of graft failure and revision ACLR. However, studies directly comparing revision ACLR rates between patients with and without gracilis tendon harvesting are lacking. Likewise, no studies to our knowledge have assessed the risk of revision surgery across all 4 HT graft configurations.

The purpose of this study was to compare anterior knee laxity, isokinetic knee extension and flexion strength, single-leg hop (SLH) test performance, subjective knee function, and the 5-year hazard of revision surgery among patients who underwent ACLR using 4 different HT graft configurations: ST3, ST4, ST-G4, and ST-G6. It was hypothesized that there would be no differences in these outcomes among the different HT graft configuration groups.

## Methods

### Participants

Ethical approval for this study was obtained from the regional ethics committee of Karolinska Institutet. The Strengthening of the Reporting of Observational Studies in Epidemiology statement was used as guidance to report this study to increase scientific quality.^43^

Patients ≥16 years without concomitant ligamentous injuries who underwent primary ACLR with an HT autograft at Capio Artro Clinic, Stockholm, Sweden, between January 1, 2005, and December 31, 2018, were evaluated for eligibility. Exclusion criteria were contralateral ACL injury or reconstruction. Patients with concomitant meniscal tears and/or cartilage injuries were not excluded.

### Surgical Technique and Rehabilitation

A single-bundle ACLR technique was performed in all cases. The ST was primarily harvested and prepared as a doubled, tripled, or quadrupled graft. In cases where the graft length or diameter was insufficient (<8 mm) or based on the surgeon’s preference, the gracilis tendon was harvested and combined to the ST graft as a doubled graft. The femoral tunnel was drilled using an anteromedial portal technique. The graft was routinely fixed on the femoral side using an EndoButton (Smith & Nephew) or a Tightrope (Arthrex) fixation device. On the tibial side, fixation was achieved with No. 2 Ethibond sutures (Ethicon) tied over an AO bicortical screw with a washer as a post. An arthroscopic all-inside technique with Fast-Fix suture anchor devices (Smith & Nephew) was used to repair meniscal tears in the posterior horn and midbody of the menisci. Tears in the anterior portion were repaired using an outside-in technique with No. 0 polydioxanone sutures (Ethicon). Irreparable tears were treated with meniscectomy.

Rehabilitation was standardized. Full weightbearing and unrestricted range of motion were permitted after isolated ACLR and ACLR with concomitant meniscal resection or meniscal repair. For patients undergoing ACLR with meniscal repair, a hinged knee brace was worn for 6 weeks. The brace was set to allow 0° to 30° of flexion during the first 2 weeks, 0° to 60° in weeks 3 and 4, and 0° to 90° in weeks 5 and 6. From the seventh week onward, the brace was discontinued, and full range of motion was encouraged. Quadriceps strengthening was limited to closed kinetic chain exercises for the first 3 months. Patients were allowed to return to sports no earlier than 6 months after ACLR, provided they met both the muscle strength and the SLH test criteria, with a limb symmetry index (LSI) of ≥90%.^11,41^

### Arthrometric Evaluation

Instrumented knee laxity was evaluated preoperatively and 6 months postoperatively at our outpatient clinic using the KT-1000 arthrometer (MEDmetric). Measurements were taken at 20° of knee flexion with a standard anterior tibial load of 30 lb (134 N). All laxity assessments were conducted by experienced sports medicine physical therapists, not blinded to the graft configuration. A minimum of 3 measurements were taken for each knee, and the median value was recorded. The side-to-side (STS) difference between the injured and healthy knee was registered. STS knee laxity was further stratified and classified into 3 groups (≤2 mm, 3-5 mm, and >5 mm) based on the International Knee Documentation Committee (IKDC) examination form.^17^

### Isokinetic Muscle Strength and SLH Test

Isokinetic concentric extension and flexion strength were measured using the Biodex System 3 (Biodex Medical Systems) 6 months postoperatively. Bilateral measurements were taken within a range of motion from 90° to 10° at an angular velocity of 90 deg/s. The uninjured contralateral leg was tested first. Before the test, patients completed a 10-minute warm-up on a stationary cycling ergometer at low resistance. They were given verbal instructions about the test and allowed 2 to 3 practice trials before the actual testing. Each leg performed 5 maximal repetitions for both extension and flexion. The highest peak torque values achieved for each leg were recorded.^11^

The SLH test was also performed 6 months postoperatively. Patients were instructed to jump forward as far as possible on 1 leg and land on the same leg. The test was considered successful only if the landing was stable. In cases of loss of balance, additional hops after landing, or an early touchdown of the contralateral leg, the hop was repeated. Before the test, patients received verbal instructions and were given several practice trials to ensure they felt confident. The test always began with the uninjured contralateral leg. Three trials were performed for each leg, and the best trial was recorded.^7,11^

Patients were considered to have achieved symmetrical isokinetic muscle strength or SLH test performance if their LSI was ≥90%.^10,11,41^

### Subjective Knee Function

Subjective knee function was assessed preoperatively, 1, 2, and 5 years postoperatively using the Knee injury and Osteoarthritis Outcome Score (KOOS), which is a commonly used patient-reported outcome measure after ACL injury and reconstruction.^32,33^ The KOOS comprises 5 subscales: Symptoms, Pain, Activities of Daily Living, Sport and Recreation, and Knee-related Quality of Life (QOL). Each subscale is scored numerically, with 0 indicating the worst possible outcome and 100 representing the best possible score. Clinically meaningful differences for each subscale are generally considered to range between 8 and 10 points.^32^

### Revision ACLR

Patients who had revision ACLR ≤5 years after their primary surgery at any institution in Sweden between January 1, 2005, and December 31, 2023, were identified using their unique Swedish personal identity number^25^ in the Swedish National Knee Ligament Registry (SNKLR).^39^

### Data Sources

Age at surgery, sex, laterality, time from injury to surgery, the presence of a medial or lateral meniscal injury or cartilage injury, medial or lateral meniscal resection or repair, graft diameter, preinjury Tegner activity level,^40^ 6-month isokinetic extension and flexion strength, and SLH test performance, as well as preoperative and 6-month KT-1000 laxity measurements were collected from our clinic database. The preoperative and postoperative KOOS were collected from the SNKLR.

### Statistical Analysis

SPSS software (Version 25.0; IBM) was used. All the variables were reported as means, standard deviations, or frequencies. All distributions were assessed for normality. Missing data were excluded from the statistical analysis for the corresponding variables. Continuous and categorical variables were compared with an analysis of variance and Pearson chi-square tests, respectively. Group differences in mean preoperative arthrometric knee laxity were analyzed with an analysis of covariance (ANCOVA) with age at surgery, sex, and time from injury to surgery as covariates.^8,9^ Group differences in mean postoperative arthrometric knee laxity were also analyzed with an ANCOVA with age at surgery, sex, time from injury to surgery, and medial meniscal repair as covariates.^8,9,14^ Differences in pre- and postoperative STS laxity, stratified according to the IKDC examination form, were analyzed using the Pearson chi-square test for trend. Mean LSI values for extension strength, flexion strength, and the SLH test were compared using an ANCOVA, adjusting for relevant covariates. For extension strength, the covariates included age at surgery, sex, and medial and lateral meniscal repair. Flexion strength was analyzed with age at surgery, sex, time from injury to surgery, and cartilage injury as covariates. The SLH test analysis was adjusted for age at surgery, sex, time from injury to surgery, medial meniscal repair, and cartilage injury. These covariates were included in the model due to observed differences between the groups and evidence from previous literature indicating their potential influence on these variables.^11^ Preoperative, as well as 1-year, 2-year, and 5-year postoperative KOOS subscale scores, were compared using an ANCOVA, with age at surgery, sex, cartilage injury (both pre- and postoperative), and medial and lateral meniscal repair (postoperative) included as covariates.^10,13,37^ A Cox regression analysis was conducted to compare the 5-year revision risk between the study groups. The ST4 group, being the most represented, served as the reference group for comparisons with the other groups. Hazard ratios were adjusted for age at surgery, sex, time from injury to surgery, cartilage injury, medial meniscal repair, lateral meniscal repair, and graft diameter.^6,31,38^ The level of significance in all analyses was 5% (2-tailed).

## Results

Of the 6007 patients assessed for eligibility, 334 were excluded due to contralateral ACL injury or reconstruction, leaving 5673 patients included. Patients were then categorized into 4 groups based on graft configuration: ST3, ST4, ST-G4, and ST-G6. The patients’ characteristics are summarized in Table 1. Significant differences were seen between the graft configuration groups for age, sex, time from injury to surgery, presence of a cartilage injury, concomitant medial meniscal or lateral meniscal repair, and graft diameter.

**Table 1:** Patient Characteristics^*a*^

	ST3	ST4	ST-G4	ST-G6	*P*
Patients, n	245	4359	915	154	
Age at surgery, y, mean ± SD	30.2 ± 10.8	30.4 ± 10.5	29.6 ± 9.8	26.8 ± 10.2	<.001^*b*^
Sex					<.001^*b*^
Male	96 (39.2)	2556 (58.5)	440 (48.0)	68 (44.2)	
Female	149 (60.8)	1803 (41.5)	475 (52.0)	86 (55.8)	
Laterality: right	126 (51.4)	2262 (51.9)	462 (50.5)	83 (53.9)	.84
Time from injury to surgery, mo, mean ± SD	20.7 ± 36.9	20.5 ± 40.8	26.6 ± 51.0	16.9 ± 25.9	<.001^*b*^
Patients with available data, n	243	4301	906	151	
MM injury	49 (20.0)	1139 (26.1)	230 (25.2)	33 (21.4)	.10
LM injury	52 (21.2)	1101 (25.3)	205 (22.4)	24 (15.6)	.17
Cartilage injury	32 (13.1)	803 (18.0)	239 (26.1)	26 (16.9)	<.001^*b*^
MM resection	30 (12.2)	698 (22.2)	160 (17.5)	19 (12.3)	.13
MM repair	10 (4.1)	345 (7.9)	41 (4.5)	11 (7.1)	.001^*b*^
LM resection	40 (16.3)	716 (16.4)	145 (15.9)	15 (9.7)	.17
LM repair	5 (2.0)	176 (4.0)	21 (2.3)	6 (3.9)	.03^*b*^
Graft diameter, mm, mean ± SD	7.8 ± 0.7	8.5 ± 0.7	8.2 ± 0.8	8.8 ± 0.8	<.001^*b*^
Patients with available data, n	245	4354	914	154	
Preinjury Tegner activity level, median (range)	7 (2-10)	7 (1-10)	7 (1-10)	7 (1-10)	.36
Patients with available data, n	224	3726	813	129	

a Data are presented as n (%) unless otherwise indicated. LM, lateral meniscus; MM, medial meniscus; ST, semitendinosus; ST-G, semitendinosus and gracilis.

b Statistically significant.

### Arthrometric Knee Laxity

Preoperatively, the groups showed no significant differences in mean STS laxity or in STS laxity values when stratified according to the IKDC knee examination form. Postoperatively, a significant difference in laxity was observed between the groups. The ST-G4 and ST-G6 groups exhibited greater mean STS laxity and increased STS laxity based on the IKDC examination form, with fewer patients showing STS laxity ≤2 mm and more patients having STS laxity between 3 and 5 mm and >5 mm (Table 2).

**Table 2:** Mean and Stratified KT-1000 Arthrometer STS Differences^*a*^

	ST3	ST4	ST-G4	ST-G6	*P*
Preoperative STS difference					
Mean ± SD, mm	3.8 ± 2.9	3.3 ± 3.1	3.5 ± 3.1	3.5 ± 3.0	.30
Group per IKDC form					
Patients with available data, n	226	3937	838	130	
≤2 mm	80 (35.4)	1643 (41.7)	301 (35.9)	47 (36.2)	.36
3-5 mm	84 (37.2)	1462 (37.1)	339 (40.5)	53 (40.8)	
>5 mm	62 (27.4)	832 (21.1)	198 (23.6)	30 (23.1)	
6-month postoperative STS difference					
Mean ± SD, mm	1.3 ± 2.1	1.5 ± 2.3	2.0 ± 2.4	1.7 ± 2.5	.001^*b*^
Group per IKDC form					
Patients with available data, n	207	3649	806	128	
≤2 mm	155 (74.9)	2579 (70.7)	478 (59.3)	78 (60.9)	<.001^*b*^
3-5 mm	46 (22.2)	908 (24.9)	268 (33.3)	43 (33.6)	
>5 mm	6 (2.9)	165 (4.5)	60 (7.4)	7 (5.5)	

a Data are presented as n (%) unless otherwise indicated. IKDC, International Knee Documentation Committee; ST, semitendinosus; ST-G, semitendinosus and gracilis; STS, side-to-side.

b Statistically significant.

### Isokinetic Muscle Strength and SLH Test

No significant differences were found between the groups in terms of mean LSI or the proportion of patients who achieved symmetrical (LSI ≥ 90%) knee extension and flexion strength or SLH test performance (Table 3).

**Table 3:** 6-Month Postoperative Side-to-Side Isokinetic Strength Measurements and Single-Leg Hop Test Results^*a*^

	ST3	ST4	ST-G4	ST-G6	*P*
Extension					
Patients with available data, n	201	3558	780	125	
LSI, %, mean ± SD	82.7 ± 16.1	84.5 ± 16.3	84.0 ± 15.9	84.4 ± 14.8	.37
LSI ≥ 90%	62 (30.8)	1274 (35.8)	274 (35.1)	48 (38.4)	.46
Flexion					
Patients with available data, n	201	3554	779	125	
LSI, %, mean ± SD	88.6 ± 14.8	89.4 ± 20.4	89.0 ± 16.3	90.4 ± 17.0	.85
LSI ≥ 90%	93 (46.3)	1632 (45.9)	341 (43.8)	61 (48.8)	.62
Single-leg hop test					
Patients with available data, n	167	2843	700	103	
LSI, %, mean ± SD	92.2 ± 14.7	92.6 ± 12.6	92.2 ± 14.0	92.4 ± 12.7	.95
LSI ≥ 90%	105 (62.9)	1939 (68.2)	481 (68.7)	68 (66.0)	.66

a Data are presented as n (%) unless otherwise indicated. LSI, limb symmetry index; ST, semitendinosus; ST-G, semitendinosus and gracilis.

### Subjective Knee Function

Preoperative KOOS subscale scores were available for 5421 patients (95.6%), while postoperative scores were recorded for 4197 patients (74.0%) at 1 year, 3000 patients (52.9%) at 2 years, and 2450 patients (43.2%) at 5 years. The only differences between the groups, though not clinically relevant (<8-10 points), were observed in the preoperative Pain and QOL subscales, as well as in the 1-year Symptoms subscale. No other significant differences between the groups were observed preoperatively or at 1, 2, or 5 years postoperatively across any of the KOOS subscales (Table 4).

**Table 4:** Preoperative and Postoperative KOOS Comparison^*a*^

	ST3	ST4	ST-G4	ST-G6	*P*
Symptoms subscale					
Preoperatively	74.2 ± 17.5 (240)	75.9 ± 17.5 (4182)	74.2 ± 18.0 (854)	77.2 ± 17.3 (145)	.05
Postoperatively, y					
1	79.0 ± 17.4 (189)	80.4 ± 16.5 (3263)	81.5 ± 16.0 (633)	77.0 ± 16.8 (112)	.03^*b*^
2	83.6 ± 14.6 (156)	82.6 ± 15.9 (2256)	81.2 ± 16.6 (517)	81.5 ± 18.5 (71)	.43
5	82.9 ± 17.7 (118)	84.7 ± 15.8 (1828)	84.4 ± 16.0 (448)	79.2 ± 18.8 (56)	.16
Pain subscale					
Preoperatively	78.9 ± 15.2 (240)	80.0 ± 15.7 (4182)	77.8 ± 16.7 (854)	81.3 ± 16.0 (144)	.005^*b*^
Postoperatively, y					
1	87.1 ± 13.0 (189)	87.8 ± 12.8 (3264)	87.2 ± 13.6 (633)	86.4 ± 12.0 (112)	.68
2	89.4 ± 10.7 (156)	88.4 ± 13.6 (2256)	86.8 ± 14.7 (517)	86.9 ± 16.7 (71)	.15
5	87.3 ± 15.7 (118)	89.7 ± 13.6 (1828)	88.9 ± 13.8 (448)	86.6 ± 17.4 (56)	.30
ADL subscale					
Preoperatively	86.3 ± 15.1 (239)	87.7 ± 14.3 (4180)	87.1 ± 14.8 (854)	87.7 ± 15.1 (144)	.38
Postoperatively, y					
1	93.4 ± 10.1 (189)	94.1 ± 10.0 (3264)	93.6 ± 11.0 (633)	94.4 ± 8.4 (112)	.61
2	95.0 ± 7.7 (156)	93.8 ± 11.3 (2256)	93.2 ± 11.7 (517)	92.3 ± 15.7 (71)	.26
5	93.2 ± 10.7 (118)	94.4 ± 10.6 (1828)	94.4 ± 10.3 (448)	91.9 ± 15.5 (56)	.23
Sport and Recreation subscale					
Preoperatively	47.5 ± 25.8 (226)	51.3 ± 27.7 (3973)	49.0 ± 25.8 (795)	51.5 ± 27.9 (139)	.11
Postoperatively, y					
1	70.5 ± 23.9 (186)	71.3 ± 23.7 (3229)	70.3 ± 24.2 (624)	71.0 ± 21.6 (109)	.90
2	75.7 ± 19.9 (153)	73.6 ± 23.9 (2231)	71.2 ± 24.6 (511)	73.5 ± 26.2 (71)	.24
5	73.7 ± 25.7 (117)	75.5 ± 24.7 (1809)	75.1 ± 24.3 (438)	72.5 ± 26.5 (54)	.88
QOL subscale					
Preoperatively	38.1 ± 22.0 (234)	40.5 ± 23.7 (4085)	36.2 ± 19.6 (825)	43.0 ± 23.8 (143)	<.001^*b*^
Postoperatively, y					
1	59.4 ± 22.0 (187)	62.7 ± 22.8 (3251)	62.9 ± 22.9 (629)	59.0 ± 21.8 (111)	.10
2	67.5 ± 21.2 (156)	67.0 ± 23.2 (2244)	65.6 ± 21.6 (513)	66.2 ± 23.0 (70)	.90
5	67.8 ± 25.0 (117)	70.7 ± 23.7 (1814)	70.4 ± 22.6 (446)	66.7 ± 25.7 (55)	.56

a Data are presented as mean ± SD (n). ADL, Activities of Daily Living; KOOS, Knee injury and Osteoarthritis Outcome Score; QOL, Quality of Life; ST, semitendinosus; ST-G, semitendinosus and gracilis.

b Statistically significant.

### Revision ACLR

The 5-year revision ACLR rates were as follows: ST4, 4.7% (207/4359); ST3, 5.3% (13/245); ST-G4, 3.7% (34/915); and ST-G6, 5.8% (9/154). The hazard of revision ACLR within 5 years of primary surgery in the ST3, ST-G4, and ST-G6 groups was not significantly different from that of the ST4 group, which served as the reference (Table 5).

**Table 5:** Revision ACLR Within 5 Years From Primary Surgery in Cox Regression Analysis^*a*^

	HR (95% CI)	*P*
ST4	1 (reference group)	
ST3	0.78 (0.43-1.39)	.40
ST-G4	0.65 (0.34-1.24)	.19
ST-G6	1.18 (0.60-2.33)	.63

a ACLR, anterior cruciate ligament reconstruction; HR, hazard ratio; ST, semitendinosus; ST-G, semitendinosus and gracilis.

## Discussion

The most important finding of this study was that knee muscle strength, SLH test performance, subjective knee function, and the hazard of revision ACLR were comparable across the different HT graft configuration groups (ST3, ST4, ST-G4, and ST-G6). However, increased anterior knee laxity at 6 months was observed in the groups with gracilis tendon harvesting (ST-G4 and ST-G6) compared with those without (ST3 and ST4).

When analyzing anterior knee laxity, no significant preoperative differences were observed among the HT graft configuration groups. At 6 months postoperatively, however, the gracilis tendon harvest groups (ST-G4 and ST-G6) exhibited—compared with the nongracilis tendon harvest groups (ST3 and ST4)—a greater mean STS laxity, a lower proportion of patients with an STS laxity ≤2 mm, and a higher proportion of patients with an STS laxity between 3 and 5 mm and >5 mm. Previous studies reported no differences in postoperative anterior knee laxity between groups with or without gracilis tendon harvest.^18,24,35,44^ These inconsistencies may be attributed to variations in sample sizes among studies. Previous research involved relatively small cohorts, whereas the present study, with over 5600 patients, may have had the statistical power to detect differences that earlier studies overlooked. HTs play an important role in controlling anterior tibial translation.^26,30,42^ However, the clinical significance of the increased anterior knee laxity in the groups with gracilis tendon harvesting remains uncertain, as it did not correlate with subjective knee function or revision ACLR.

Knee flexion strength deficits are a common occurrence after ACLR with HT autografts. A recent randomized controlled trial^12^ found that flexion strength deficits persist for up to 2 years after ACLR using HT autografts (both ST and gracilis). This provides the rationale for gracilis-sparing techniques with the aim of minimizing knee flexion strength deficits after ACLR.^24,34,44^ Symmetrical knee strength is considered important for returning to sport after ACLR^11,41^ and is linked to a reduced risk of reinjury.^16,23^ Regeneration of the ST tendon occurs in the majority of patients who undergo ACLR.^15,27,36^ However, there are uncertainties about whether regeneration occurs at the anatomic tendon insertion and the functional capacity of the regenerated tissue.^3^ Janssen et al^19^ assessed with magnetic resonance imaging 22 patients who underwent ACLR with HT autografts and reported that the regenerated tendons had no significant effect on isometric or isokinetic knee flexion strength. However, the literature reports conflicting results regarding the effects of gracilis sparing on knee flexion strength after ACLR. Some studies have reported greater knee flexion strength deficits after gracilis harvesting,^18,21,24,34^ while others found no effect of gracilis harvesting on knee flexion strength.^5,7,20,29^ Due to heterogeneity across studies in variables such as rehabilitation protocol, isokinetic versus isometric contraction, and the angular velocity used for isokinetic testing, making direct comparisons is challenging. The present study found no differences in peak isokinetic knee flexion torque (measured at 90 deg/s) between ST and ST-G techniques. It can be hypothesized that gracilis harvesting does not significantly affect knee flexion strength or that the semimembranosus and biceps femoris muscles effectively compensate for the loss of the gracilis tendon. On the other hand, it is also possible that the benefits of gracilis preservation on knee flexion strength may not be fully captured by isokinetic testing, as the gracilis tendon primarily contributes to force generation at higher degrees of knee flexion.^1^ In contrast, in the present study, isokinetic testing was performed in a seated position with a maximal flexion of 90°. However, sports activities that require high degrees of knee flexion are limited, and the potential advantages of increased knee flexion strength at these angles remain uncertain.^35^

Subjective knee function was similar across all study groups. Statistically significant, yet clinically irrelevant, differences (<8-10 points) were observed in the preoperative Pain and QOL subscales, as well as in the 1-year Symptoms subscale. No other differences between the groups were noted preoperatively or at 1, 2, or 5 years postoperatively across any of the KOOS subscales. This suggests that all 4 HT graft configurations (ST3, ST4, ST-G4, and ST-G6) result in comparable outcomes in subjective knee function after ACLR and that gracilis tendon harvesting does not significantly affect subjective knee function.

An important finding of this study was that the 5-year revision ACLR rates were similar across all 4 HT graft configuration groups (ST4, 4.7%; ST3, 5.3%; ST-G4, 3.7%; and ST-G6, 5.8%). Indeed, the hazard of revision in the ST3, ST-G4, and ST-G6 groups did not differ significantly from that of the ST4 group, which served as the reference. These findings have 2 important implications: (1) that all HT graft configurations, regardless of the number of strands, are safe and yield consistent, comparable outcomes in terms of revision surgery; and (2) although several studies have shown that the hamstring muscles help unload the ACL by controlling anterior tibial translation and internal tibial rotation,^4,26,28^ gracilis tendon harvesting does not significantly affect the hazard of revision surgery. Furthermore, the increased postoperative knee laxity observed in the gracilis-harvesting groups (ST-G4 and ST-G6) did not correlate with an increased hazard of revision ACLR, making its clinical significance uncertain.

### Strengths and Limitations

This study has several strengths, including the analysis of a large cohort of 5673 patients and the comparison of multiple HT graft configurations (ST3, ST4, ST-G4, and ST-G6). To our knowledge, this is the first study to conduct such a comparison. Additionally, the study evaluated several outcomes, including anterior knee laxity, isokinetic knee flexion and extension strength, subjective knee function, and revision surgery. Finally, all surgeries, laxity assessments, and isokinetic strength measurements were conducted at the same institution.

Several limitations should be acknowledged. First, interobserver variability in arthrometric laxity assessment is often cited as a potential limitation in studies using the KT-1000 arthrometer. However, all physical therapists involved in the assessments were specialized in sports medicine and had extensive experience with arthrometric laxity testing. Furthermore, the large cohort included helped minimize the potential effect of this limitation. Second, preoperative isokinetic knee strength was not assessed, as at our institution muscle strength is routinely measured only postoperatively. Any potential differences in preoperative strength between the groups could have influenced the postoperative strength outcomes. Third, the 2- and 5-year KOOS follow-up rates were suboptimal (52.9% and 43.2%, respectively), which is a common issue in large cohort registry studies.^7,10,13,22^ However, KOOS subscale scores were still available for 3000 and 2450 patients at the 2- and 5-year marks, respectively. These large sample sizes provide high statistical power and enhance the generalizability of the results. Fourth, graft configuration may, in some cases, have been surgeon dependent. Although the general guideline was to harvest the gracilis tendon only when the ST diameter was deemed insufficient (<8 mm), surgeons had the discretion to use an ST+G configuration regardless of this criterion. Fifth, the outcome assessed in this study was revision ACLR rather than graft rupture, as this information is not available in our registry. Therefore, potential differences in graft rupture rates between the study groups could not be evaluated. Finally, data regarding return to sport or activity level after ACLR were not available. Any potential differences in these factors across the groups could have influenced the hazard of revision ACLR. However, the preinjury Tegner score did not show significant differences between the study groups, and any differences in return to sport or activity level post-ACLR would likely have affected all groups similarly, without introducing systematic bias.

## Conclusion

All 4 HT graft configurations (ST3, ST4, ST-G4, and ST-G6) yielded comparable outcomes in isokinetic knee flexion and extension strength, SLH test performance, subjective knee function, and the hazard of revision surgery after ACLR. The groups that underwent gracilis tendon harvesting (ST-G4 and ST-G6) exhibited increased anterior knee laxity at 6 months compared with the groups that did not (ST3 and ST4). However, the clinical significance of this finding remains uncertain, as the greater knee laxity did not correlate with subjective knee function or revision ACLR.
